# Heparin solution in the prevention of occlusions in Hickman^®^ catheters a randomized clinical trial*

**DOI:** 10.1590/1518-8345.3310.3385

**Published:** 2021-01-08

**Authors:** Sandra Regina da Silva, Mitzy Tannia Reichembach, Letícia Pontes, Gisele de Paula e Silva Carneiro Mendes de Souza, Solena Kusma

**Affiliations:** 1 Universidade Federal do Paraná, Complexo Hospital de Clínicas, Curitiba, PR, Brazil.; 2 Universidade Federal do Paraná, Curitiba, PR, Brazil.; 3 Universidade Federal do Paraná, Departamento de Saúde Coletiva, Curitiba, PR, Brazil.

**Keywords:** Evidence-Based Nursing, Randomized Controlled Trial, Central Venous Catheters, Catheter Obstruction, Heparin, Sodium Chloride, Enfermagem Baseada em Evidências, Ensaio Clínico Controlado Randomizado, Cateteres Venosos Centrais, Obstrução do Cateter, Heparina, Cloreto de Sódio, Enfermería Basada en la Evidencia, Ensayo Clínico Controlado Aleatorio, Catéteres Venosos Centrales, Obstrucción del Catéter, Heparina, Cloruro de Sodio

## Abstract

**Objective:**

to evaluate the effectiveness of the 50 IU/mL heparin solution compared to the 0.9% isotonic saline solution in preventing occlusion of the double lumen Hickman^®^ catheter, 7 and 9 French, in patients undergoing hematopoietic stem cell transplantation.

**Method:**

a triple-blind randomized clinical trial. 17 double-lumen catheters (heparin group: n=7 and 0.9% isotonic saline group: n=10) were analyzed in which the two catheter routes were evaluated separately, totaling 34 lumens. The outcome variables were *occlusion without reflux* and *complete occlusion*. Descriptive analyses were performed using the Chi-square test and, of survival, according to the Kaplan-Meier test.

**Results:**

the mean number of days until the occlusion outcome was 52 in the heparin group and 13.46 in the 0.9% isotonic saline group in the white catheter route (p<0.001). In the red route, the mean follow-up days in the heparin group were 35.29, with no occlusion and 22.30 in the 0.9% isotonic saline group until the first occlusion (p=0.030).

**Conclusion:**

blocking with 50 IU/mL heparin solution is more effective than 0.9% isotonic saline in preventing occlusion of the Hickman^®^ catheter. Brazilian Registry of Clinical Trials: RBR-3ht499.

## Abstract (pt)

**Objetivo:**

avaliar a efetividade da solução de heparina 50 UI/mL comparada à solução salina isotônica 0,9% na prevenção de oclusão do Cateter de Hickman^®^ duplo lúmen, 7 e 9 *french*, em pacientes submetidos ao transplante de células-tronco hematopoéticas.

**Método:**

ensaio clínico randomizado triplo cego. Foram analisados 17 cateteres duplo lúmen (grupo heparina n=7 e grupo solução salina isotônica 0,9% n=10) nos quais as duas vias do cateter foram avaliadas separadamente, totalizando 34 lúmens. As variáveis de desfecho foram *oclusão sem refluxo* e *oclusão completa*. As análises descritivas foram realizadas mediante o teste Qui-quadrado e, de sobrevida, sob o teste de Kaplan-Meier.

**Resultados:**

a média de dias até o desfecho oclusão foi de 52 no grupo heparina e de 13,46 no grupo solução salina isotônica 0,9% na via branca do cateter (p<0,001). Na via vermelha, a média de dias de acompanhamento do grupo heparina foi de 35,29, sem ocorrência de oclusão, e de 22,30 no grupo solução salina isotônica 0,9% até a primeira oclusão (p=0,030).

**Conclusão:**

o bloqueio com solução de heparina 50 UI/mL é mais efetivo em relação à solução salina isotônica 0,9% na prevenção da oclusão do Cateter de Hickman^®^. Registro Brasileiro de Ensaios Clínicos: RBR-3ht499.

## Abstract (es)

**Objetivo:**

evaluar la eficacia de la solución de heparina 50 UI/mL comparada con la solución salina isotónica al 0,9% para prevenir oclusiones en catéteres de Hickman^®^ doble lumen, 7 y 9 *French,* en pacientes sometidos a trasplante de células madre hematopoyéticas.

**Método:**

ensayo clínico aleatorizado triple ciego. Se analizaron 17 catéteres de doble lumen (grupo de heparina: n=7 y grupo de solución salina isotónica al 0,9%: n=10) en los que se evaluaron por separado las dos vías del catéter, totalizando 34 lúmenes. Las variables de resultado fueron *oclusión sin reflujo* y *oclusión completa*. Los análisis descriptivos se realizaron mediante el test de Chi-cuadrado y, los de sobrevida, con el test de Kaplan-Meier.

**Resultados:**

la media de días hasta el resultado de oclusión fue de 52 en el grupo de heparina y de 13,46 en el grupo de la solución salina isotónica al 0,9% en la vía blanca del catéter (p<0,001). En la vía roja, la media de días de seguimiento del grupo de heparina fue de 35,29 sin oclusión y de 22,30 en el del grupo solución salina isotónica al 0,9% hasta la primera oclusión (p=0,030).

**Conclusión:**

el bloqueo con solución de heparina 50 UI/mL es más eficaz en relación con la solución salina isotónica al 0,9% para prevenir oclusiones en catéteres de Hickman^®^. Registro Brasileño de Ensayos Clínicos: RBR 3ht499.

## Introduction

The Central Venous Catheter (CVC) is a device used for intravenous fluid infusion and blood extraction, the tip of which is positioned in the superior or inferior vena cava ^(^ 1 ^)^. This type of catheter should be selected from the assessment of the patient’s needs, such as the condition of the venous network, the treatment regime and time, in addition to the technical capacity of the team that handles the device ^(^ 2 ^)^.

CVC are classified as short- and long-term. Short-term CVC are those who are inserted by direct venipuncture, with a permanence time of less than one month. Long-term ones are used for patients with an indication for treatment longer than 21 days^(^ 2 ^-^ 3 ^)^. These are divided into totally implanted central venous catheter (CVC-TI) and semi-implanted central venous catheter (CVC-SI) and are indicated for cancer patients, and for those with hydroelectrolytic disorders, malnutrition, renal failure and acquired immunodeficiency syndrome. Among them, the Hickman^®^ catheter^(^ 3 ^)^, characterized as a CVC-SI, has benefited patients undergoing Hematopoietic Stem Cell Transplantation (HSCT).

HSCT is a therapy used for malignant and non-malignant hematological diseases, which aims to replace the deficient bone marrow with a healthy one^(^ 4 ^)^. The use of the CVC-SI in this population is mainly due to the intense and continuous infusion of hydration, drugs, blood components and parenteral nutrition, necessary for this therapy. However, although the use of CVC-SI in HSCT is extremely favorable, it is not without complications^(^ 5 ^)^.

In a study that investigated incidents related to CVC-SI in patients undergoing HSCT, it was concluded that occlusion was the preponderant event in relation to other possible complications with the Hickman^®^ catheter^(^ 6 ^)^. The occlusion of a CVC is a worrying event and it frequently requires the interruption of therapy or, still, the patient’s exposure to a new invasive procedure^(^ 1 ^)^.

Permeability is the ideal condition of a CVC that consists of the act of infusing fluids and collecting blood from this device without resistance. Occlusion is characterized by the permeability dysfunction of this device and can be classified into three degrees: partial, without reflux, and complete. The first is defined as resistance to infusion or slow reflux. Occlusion without reflux presents an inability to obtain blood reflux, but in a condition of infusion without resistance^(^ 2 ^)^ and complete occlusion is defined as the impossibility of infusion and reflux in the CVC.

The causes can be mechanical, chemical or thrombotic^(^ 1 ^)^. Chemical and thrombotic occlusions are preventable, as long as the recommended techniques for flush and blocking the device^(^ 1 ^)^ are maintained, in addition to the appropriate blocking solution for preventing occlusion, that is, for guaranteeing permeability^(^ 2 ^,^ 7 ^)^.

Regarding the maintenance of venous devices in general, there is already a consensus on the use of 0.9% Isotonic Saline Solution (ISS) for flush. However, for the long-term solution of CVC blockade, there is no strong clinical evidence for the ISS recommendation on the heparin solution in reducing the incidence of occlusion^(^ 5 ^,^ 7 ^-^ 9 ^)^.

It is highlighted that, in the available studies, there is heterogeneity both due to the different types of catheter and research protocol, as well as to the basic pathology of the patient, in addition to the variation in the nursing practice, when comparing the frequency of flush, the concentration of heparin and the volume to be administered in the different services.

A number of studies recommend the development of clinical trials comparing different concentrations of heparin with ISS in more homogeneous samples^(^ 7 ^-^ 8 ^,^ 10 ^)^. In this context, the present study hypothesized that the use of a 50 IU/mL (International Units/milliliter) heparin solution to block the Hickman^®^ catheter is more effective in preventing occlusion when compared to ISS. Thus, the objective was to evaluate the effectiveness of the 50 IU/mL heparin solution compared to isotonic saline solution in preventing occlusion of the double lumen Hickman^®^ catheter, 7 and 9 French, in patients undergoing hematopoietic stem cell transplantation.

## Method

This is a randomized triple-blind clinical trial performed at the Bone Marrow Transplant Service (BMTS) - Inpatient Unit of a tertiary-level public teaching hospital in Curitiba-PR, which performs autologous and allogeneic HSCT. For each type of allogeneic HSCT, there is a different time for an adequate response of the new graft, a mean of 21 days and, in the event of complications, the hospital stay can be prolonged. The BMTS - Inpatient Unit is composed of a multi-professional team, where the BMTS nursing team is divided into 35 nurses, seven nursing technicians and four nursing assistants, totaling 46 nursing professionals. This service has 24 beds distributed equally among adults and pediatrics to perform HSCT, where only 13 beds are active. It is noteworthy that, during the collection period, the service capacity was reduced from 13 to ten beds, with a mean of six HSCT/month.

The participants were recruited for research after admission, prior to insertion of the Hickman^®^ catheter by one of the researchers. The inclusion criteria were defined as follows: patients children and adults, admitted to the BMTS undergoing HSCT at the service, with the implantation of the Hickman^®^ catheter in the hospital, which was the research field. The exclusion criteria were the following: being on anticoagulant by any route of administration, being on fibrinolytic therapy, presenting a history of allergy to heparin components and being admitted to another unit after implantation of the catheter.

Those who met the inclusion criteria and agreed to participate in the study were asked to sign the Free and Informed Consent Form (FICF), the Legal Responsible Free and Informed Consent Form (LRFICF), as well as the Free and Informed Assent Form (FIAF) for children aged seven to 12 years old.

After inserting the Hickman^®^ catheter in the Surgical Center, the surgeon and the nursing team in the sector forwarded the cut end of the catheter to the BMTS, for the measurement and definition of an adequate volume for the block, using the following adapted formula: *priming volume = reduced length (cm) ÷ total length (cm) x total priming volume (+20%)* ^(^ 2 ^)^.

The randomization process was carried out by a nurse external to the research, PhD in Nursing from the Federal University of Paraná, who used the Random computerized system to generate a list of random order of solution allocations^(^ 11 ^)^. Each coded card was stored in sequential opaque envelopes that were sealed. These envelopes were then handed over to the main researcher. Each envelope was opened as the participants were included in the survey, ensuring that the increasing order of opening of the envelopes was maintained.

The contact to communicate the inclusion of the participant and the code of the solution to be prepared was made through the WhatsApp^®^ application between the main researcher and the pharmaceutical company. The envelope containing the information of which solution corresponded to code A and to code B was under the responsibility of the pharmaceutical company and was only revealed to the researchers after analyzing the data. The intervention group was considered the unfractionated heparin solution of porcine origin diluted in ISS, with a final concentration of 50 IU/mL, and the ISS control group.

The participants who met the inclusion criteria were randomLy divided into blocks of six between the two research groups: three for the intervention group and three for the control group. As included in the research, the participants received a code for the solution to be used and the syringes were coded. The syringes were filled with blocking solutions at the hospital’s pharmacy service and four 10 mL syringes *per* participant/day were filled and coded (according to randomization) with 3 mL of solution in each. These syringes were delivered to the service daily and remained stored in the refrigerator for a period of 24 hours. The solutions were visually identical, both transparent, and the nurse and/or nursing technician considered only the corresponding syringe code for each participant, according to randomization.

A convenience sample was used, which includes consecutively accessible participants, for a period of time, that meet the eligibility criteria. Data collection took place between March 22 and September 22, 2017, totaling 180 days, with a sample of 17 catheters. The catheter follow-up period until the outcomes ranged from three to 57 days.

Data collection took place using two pre-elaborated instruments: one for the registration of sociodemographic, clinical, and catheter-related variables and another for the daily collection of the catheter permeability conditions.

The team responsible for data collection was composed of the main researcher and of ten nurses who agreed to participate in data collection. The forms filled out by the assistance team were collected daily and later tabulated on an Excel^®^ 2007 electronic spreadsheet.

Prior to data collection, the nursing team responsible for blocking the catheter, using heparin 50 IU/mL and ISS solutions, was instructed on the recommended actions for maintaining the catheter permeability (flush that precedes the block and the block properly). This allowed for the standardization of procedures related to blocking and opening the lumens of the Hickman^®^ catheter.

The catheter was released for use first by confirming the correct position of the catheter tip after chest X-ray, followed by a positive catheter permeability test, which consists of blood aspiration and ISS infusion without resistance. In catheters permeable to this first assessment, control and recording of the catheter’s performance was initiated until the outcomes.

**Figure 1:**
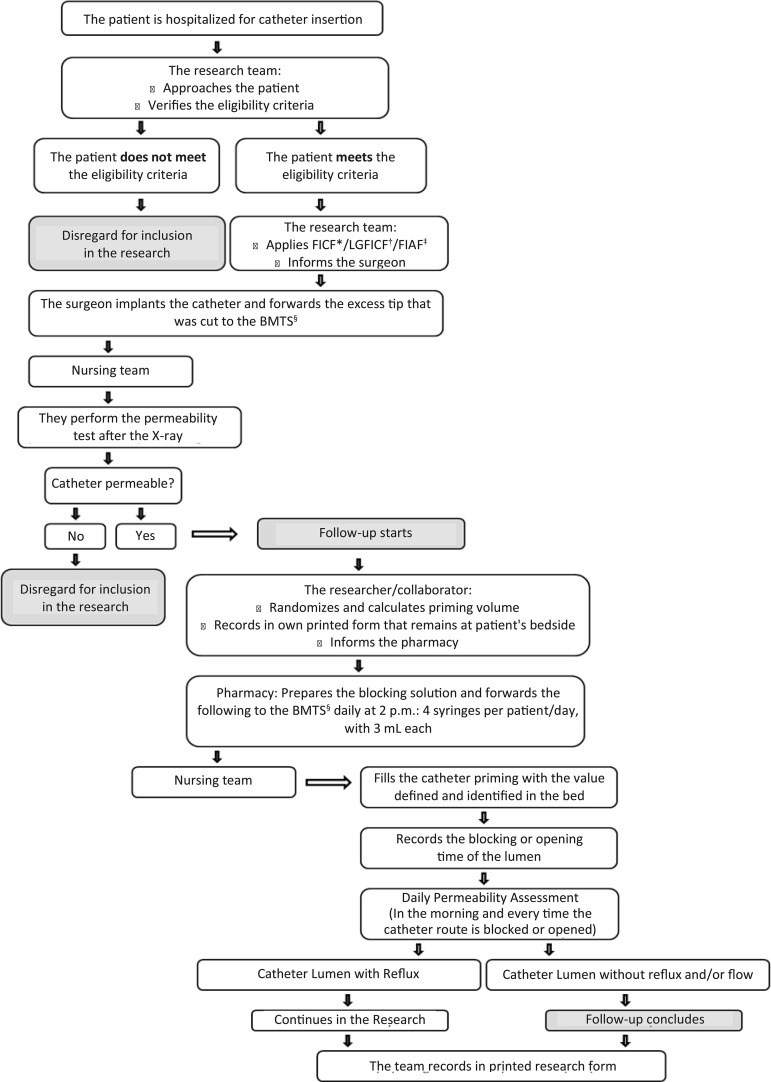
Flow diagram of the inclusion of participants in the research. Curitiba, PR, Brazil, 2017 ^*^FICF = Free and Informed Consent Form; ^†^LRFICF = Legal Responsible Free and Informed Consent Form; ^‡^FIAF = Free and Informed Assent Form; ^§^BMTS = Bone Marrow Transplant Service; ^ǁ^mL = milliliter

The nursing team had the functions of filling the catheter priming with the defined volume, registered in the bed identification; recording the time for blocking and/or opening the road; performing the permeability assessment according to the protocol and registering in the research form. The flow diagram of these activities is shown in Figure 1.

*Catheter maintenance protocol*: in order to preserve the catheter’s permeability, the practice of flush and blocking was maintained according to pre-established criteria, namely: a) *blocking of catheter in use*: flush with ISS + start stop + positive pressure technique, prior to the injection of the blocking solution; b) *unblocking the catheter*: blood aspiration of 1 mL for adults and of 0.5 mL for children, followed by flush with ISS + start stop + positive pressure^(^ 2 ^)^. After this procedure, the lumen was used for fluid infusion or blocked again.

**Figure 2:**
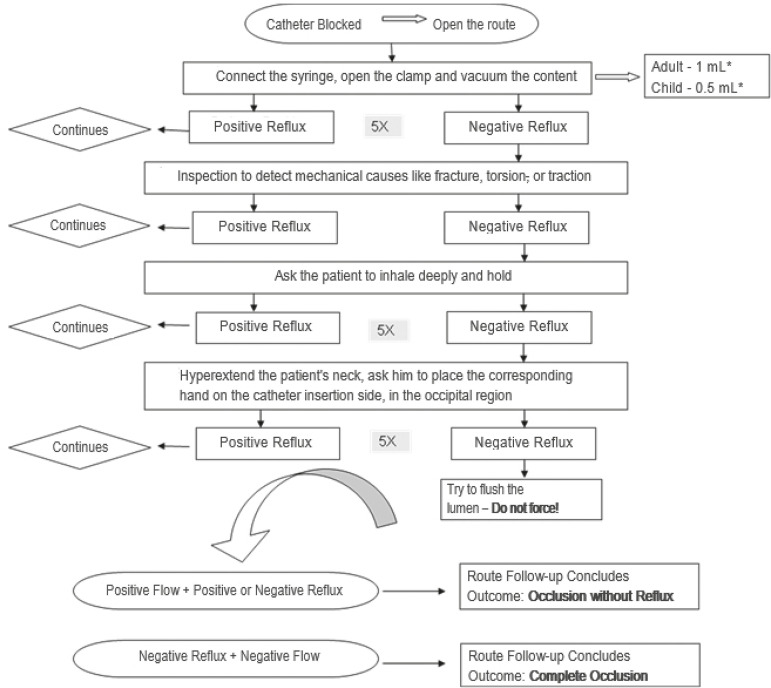
Catheter permeability assessment protocol. Curitiba, PR, Brazil, 2017 ^*^mL - milliliter

*Protocol for assessing the permeability of the catheter*: to determine if the reflux is positive, the catheter pathway was opened and with aspiration of the previously defined intraluminal content (1 mL/adult and 0.5 mL/child) in up to five attempts. With the return of blood, adequate reflux was considered. In the absence of blood return, other maneuvers were performed, which should be successful in up to three steps: *1)* inspecting mechanical causes, such as fracture, torsion or traction; *2)* asking the patient to inhale and hold the air (up to five attempts); *3)* hyperextending the patient’s neck and asking him to place the corresponding hand next to the catheter insertion in the occipital region (up to five attempts) (Figure 2).

In cases in which blood return was not successful, after the four steps described, the catheter was flushed *without forcing*. And, when there was no difficulty in infusing ISS into the catheter, it was called *occlusion without reflux*, characterized by the inability to draw blood, but in an infusion condition without resistance^(^ 2 ^)^. *Occlusion without reflux* was also considered when blood reflux was positive only after flush with ISS.

When flushing the route, after the four attempts, there was resistance to infuse ISS, it was called *complete occlusion*, characterized by the impossibility of infusion or aspiration in the CVC^(^ 2 ^)^.

In both cases, monitoring of the occluded catheter route by the research team was terminated. In complete occlusion, the clearance procedure established in the service was carried out.

Considering that in the Hickman^®^ catheter the catheter routes are independent, when the monitoring of one of the routes due to occlusion is closed, the other remained in the research until the outcome.

*Statistical analysis of the data*: the data resulting from the research were divided into two groups for description and comparison, namely: group A and group B. They were typed and tabulated in Microsoft Excel^®^ 2007 spreadsheets and later analyzed with the help of the Statistical Package for the Social Sciences (SPSS^®^) program, version 22.

The characteristics of the participants were compared using contingency tables, by means of the Chi-square test. In the survival analysis, the date of insertion of the catheter was considered to start the follow-up until the occlusion occurred. The Kaplan-Meier test was performed and the survival curves were compared (log rank test) to determine if there were differences in the distribution of occurrence of the occlusion event between the two types of blocking solutions. In all the tests, a significance level of 5% was considered.

This study was preceded by the approval of the Research Ethics Committee (REC) of the Clinical Hospital Complex of the Federal University of Paraná, under the consubstantiated opinion No. 1,967,302 and is registered in the Brazilian Registry of Clinical Trials (*Registro Brasileiro de Ensaios Clínicos*, ReBEC) under No. RBR-3ht499.

## Results

**Figure 3:**
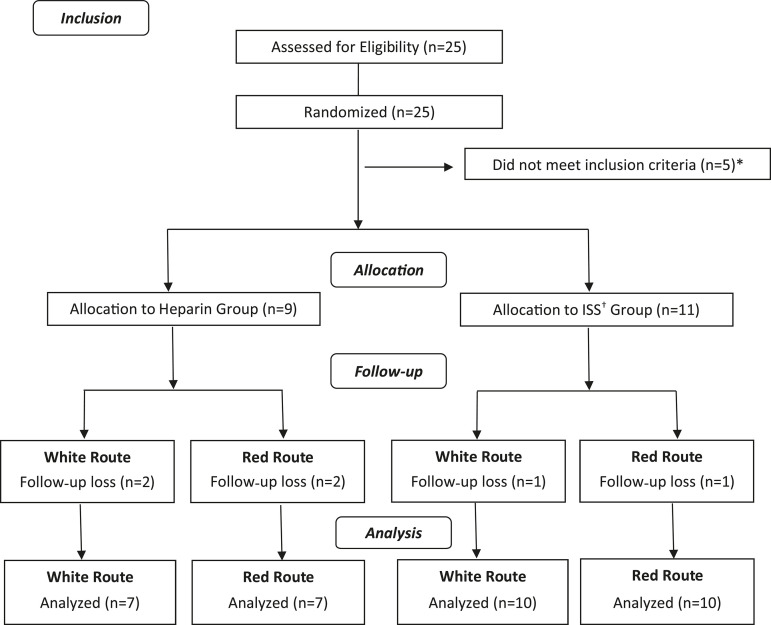
Flowchart of inclusion, randomization, and analysis of the groups according to the statement of the Consolidated Standards of Reporting Trials. Curitiba, PR, Brazil, 2017 ^*^(n=5) = Five catheters were excluded from the analysis, 4 Leonard^®^ and 1 Broviac^®^ CVC-SIs; ^†^ISS = 0.9% isotonic saline solution

A total of 25 CVC-SI were eligible for the research (Figure 3). Of these, four were Leonard^®^ CVC-SI and one, a Broviac^®^ CVC-SI, which were excluded from the analysis. There were also three follow-up breaks: two in the heparin group and one in the ISS group. The reasons for follow-up failure were the following: insertion of the catheter for infusion of total parenteral nutrition, for follow-up in another unit; a follow-up was interrupted due to lack of chemotherapy to start treatment and a catheter was no longer followed-up due to difficulties with the donor, which resulted in early discharge in these three situations. Thus, 17 catheters were analyzed, ten from the ISS group and seven from the heparin group. The two catheter routes were evaluated separately, totaling 34 lumens.

The exclusion of the catheters occurred after randomization, due to the fact that, although all the patients undergoing HSCT in the BMTS undergo the implantation of a Hickman^®^ type CVC-SI, during the data collection period, the insertion of other types of catheter had some bearing, either due to lack of Hickman^®^ catheters in the service or to the difficulty of appropriate venous access.

Regarding the sociodemographic variables of the participants, male patients predominated and, among the diagnoses, so did congenital diseases, with 71.40% in the heparin group and 50% in the ISS group. Patients under the age of 18 years old represented 71.42% of the heparin group and, in the ISS group, they had the same incidence between adults and children under 18 years old. The most used insertion site was the right internal jugular vein in both groups. The analyses that involved the occlusion outcome with sociodemographic variables, clinical variables, and those related to the catheter caliber did not show statistical significance.

The catheter routes were analyzed separately (white route and red route), with a view to infusing different solutions in both. The red route, of larger caliber, is used for blood collection or infusion of blood components.

Among the outcomes of the white catheter route, in the heparin group there was complete occlusion (14.28%). In the ISS group, there were nine occlusions, three complete (30%) and six without reflux (60%), showing a significant difference between the groups (p=0.006).

Among the outcomes of the red catheter route, there was no occlusion in the heparin group. In the ISS group, five occlusions occurred: one complete (10%) and four without reflux (40%).

**Figure 4:**
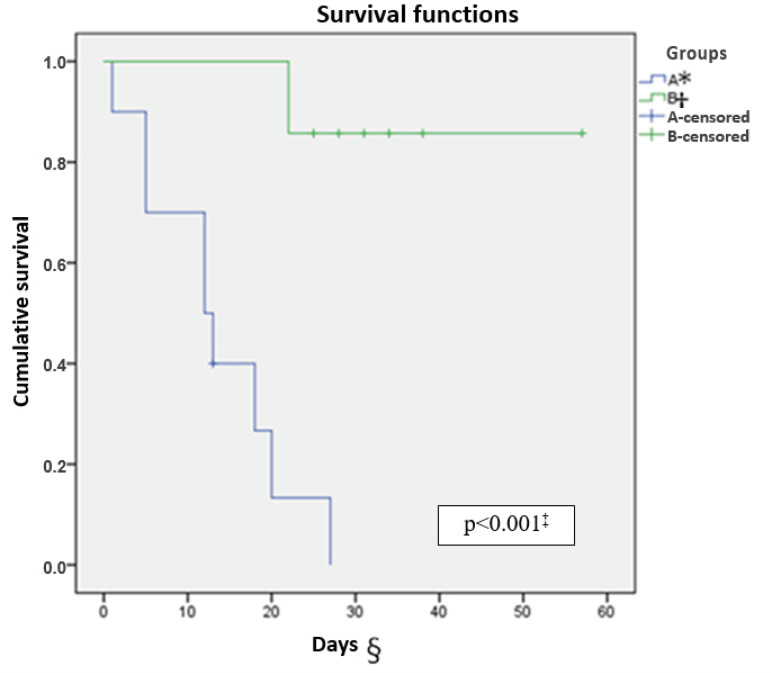
Survival curve of the white catheter route. Curitiba, PR, Brazil, 2017 ^*^A = 0.9% isotonic saline group; ^†^B = Heparin group; ^‡^p<0.001 - Log Rank (Mantel-Cox); ^§^Days = Survival curve in days

**Figure 5:**
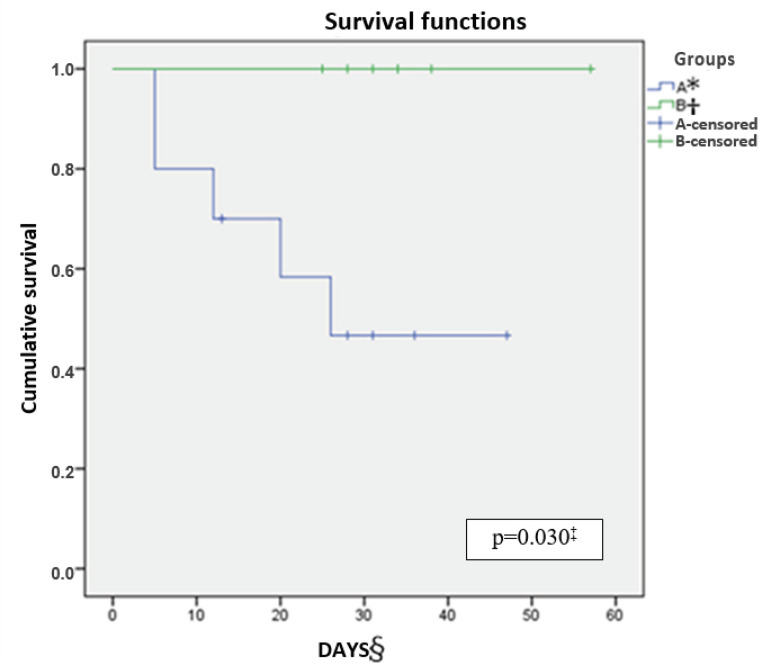
Survival curve of the red catheter route. Curitiba, PR, Brazil, 2017 ^*^A = 0.9% isotonic saline group; ^†^B = Heparin group; ^‡^p = 0.030 - Log Rank (Mantel-Cox); ^§^Days = Survival curve in days

Figures 4 and 5 demonstrate, respectively, the survival curves (days) of permeability of the white and red routes of the catheter until the occurrence of the occlusion event in each group. The mean number of days until the occlusion outcome was 52 in the heparin group and 13.46 in the ISS group on the white catheter route (p<0.001). In the red catheter route, the general follow-up mean was 35.29 days in the heparin group and there was no occlusion and 22.30 days until the occurrence of the occlusion event in the ISS group (p=0.030).

## Discussion

When assessing permeability, this study considered blockade as the condition of a closed catheter with solution inside the lumen^(^ 2 ^)^ and the analysis of the permeability of the catheter lumens occurred at its opening. This research allowed us to identify that the mean number of days until the occlusion outcome was greater in the heparin group in the two catheter routes (white and red), with p<0.001 and p=0.030, respectively.

Other studies show similar results, despite the difference in the concentration of heparin, which ranged from 10 to 5,000 IU/mL, used for blocking, over time to assess the permeability and type of the catheter. This heterogeneity is confirmed in a systematic review, which verified this variety of actions^(^ 8 ^)^.

German researchers performed triple-lumen CVC blocking with 5000 IU/mL heparin solution, a concentration that exceeds current recommendations, versus ISS flush and 200 mg/mL vitamin C flush and considered occlusion without reflux as the primary outcome, concluding there is a difference between the use of heparin and ISS (p <0.04), choosing the heparin solution, more effective than ISS in maintaining the permeability of the analyzed CVC^(^ 13 ^)^. Regarding the use of heparin solution to block the catheter, when used in high concentration, although this practice has benefits in maintaining CVC permeability, it is important to remember that it is a drug and, as such, it deserves attention in its use. There are also concerns about the cost of heparin versus ISS^(^ 14 ^)^.

Another study, when comparing the 200 IU/mL heparin flush versus ISS in Broviac-Hickman CVC, defined partial, without reflux and complete occlusion as the outcome. The results show that the incidence of occlusion was significantly higher in the ISS group (82.17%) compared to the heparin group (40.19%) (p=0.0002)^(^ 15 ^)^.

The 100 IU/mL heparin solution in 5 mL was compared to ISS to maintain the permeability of CVC in another study and no difference was identified between the use of the solutions (p=0.744)^(^ 16 ^)^. It is worth highlighting that the heparin concentration of 100 IU/mL is the maximum recommended concentration^(^ 7 ^)^ and that the volume of 5 mL far exceeds what is necessary to fill the priming of a CVC. This result differs from the present study, especially in relation to the blocking solution, which used ISS versus 50 IU/mL heparin, with a volume variation between 0.4 to 1 mL to fill the priming, respecting the international recommendation for the heparin concentration and that of the manufacturer for the lumen filling volume^(^ 7 ^)^, which minimizes the risk of the heparin solution contacting the patient’s bloodstream, ensuring greater safety for this practice.

Other studies with different concentrations of heparin solution (10 IU/mL and 100 IU/mL), which were also compared with ISS, found no difference between the solutions in maintaining the permeability of short-term CVC, in adults^(^ 16 ^-^ 17 ^)^.

An Iranian study evaluated complete occlusion and occlusion without reflux. Flush was compared with 10 mL of ISS versus 10 IU/mL heparin flush, using 3 mL of the solutions after the injection of each medication. Researchers claim to have found no significant difference in relation to the two types of occlusion assessed between the use of ISS and heparin^(^ 18 ^)^. The use of the heparin solution after each injection of a drug should be carefully evaluated, considering that a patient may have several drugs in a 24-hour period, such as, for example, those undergoing complex treatments like HSCT.

In the case of a long-term catheter, a retrospective study compared the efficacy of ISS with a 100 IU/mL heparin solution in CVC-TIs and the three types of occlusion were analyzed. The results show that there is no significant difference between the groups regarding the three types of occlusion (p=0.11)^(^ 14 ^)^. Another study, which also evaluated CVC-TI and aimed to assess occlusion without reflux, compared flush with 10 mL of ISS to flush with 10 mL of ISS + 300 IU heparin (3 mL). The results do not show significant differences in catheter-free survival^(^ 19 ^)^, as well as the study that evaluated complete occlusion with ISS block versus block with 500 IU/10 mL heparin solution^(^ 20 ^)^.

There is limited evidence regarding the comparison of using ISS and heparin in terms of efficacy or safety^(^ 7 ^-^ 10 ^,^ 21 ^)^. The importance of the CVC blocking practice with an adequate solution is justified by the risk of infection due to the formation of a fibrin network and to the adherence of bacteria and fungi with loss of permeability^(^ 22 ^)^. In addition to the risk of infection, the occlusion of the catheter can lead to its early withdrawal, exposing the patient to the risk of a new surgical procedure^(^ 6 ^)^ plus the risk to his safety, since a second catheter is certainly inserted at a more unfavorable moment, within the HSCT process.

The results found in this study, that is, the better performance of the heparin solution in preventing occlusion of the Hickman^®^ catheter, favor the use of heparin, considering its low concentration and the volume of solution infused that varied from 0.4 to 1 mL. Maintaining the permeability of the CVCs in these patients is crucial, in view of the high risk of inserting a second venous device, in addition to the cost for the institution of performing the procedure for a new catheter.

It is also believed that, in addition to the most effective blocking solution, the adequate performance of flushes, positive pressure blocking and volume according to the actual size of the catheter in the patient, will favor the maintenance of that device permeability associated with patient safety.

There are limitations both in the execution and in data analysis. With regard to execution, it is emphasized that the performance of HSCT depends on the combination of factors between the patient’s clinical status, the condition of the donor and the availability of a bed in the BMTS. During the development of this research, it was sometimes found that, due to the patient’s clinical condition and to the donor’s conditions, it was feasible to perform the transplant, but the unavailability of a bed in the service required that, after the catheter was implanted, its maintenance was the responsibility of other units until the patient was admitted to the BMTS, which configured an exclusion criterion.

The main causes for this occurrence were the following: lack of bed and/or professionals to assist the patient in the BMTS and reduction in the number of procedures in the operating room due to the lack of available anesthesiologists, reducing the sample size due to the impossibility of recruiting all the eligible patients. There was also a reduction in the number of active beds in the BMTS, in April 2017, from 13 to 10.

Another limitation for the execution is related to the lack of important inputs to carry out the research, such as the Hickman^®^ catheter and 10 mL syringe, the latter defined as the first choice for flushes with ISS before blocking and for blocking solutions. In the absence of this input, 20 mL syringes with ISS were used for flush before blocking, seeking to respect the recommendation of using syringes that promote less positive pressure in the catheter^(^ 2 ^)^. For the blocking solution, due to the reduced volume, the 5 mL syringe was used.

In relation to the limitations for analysis and discussion, the following are highlighted: the withdrawal of five catheters for analysis, as they present some characteristics that differ from the ones of the Hickman^®^ catheter, which reduced the sample size; the difference in the outcomes listed in other studies, considering that the occlusion outcome, classified as partial occlusion, occlusion without reflux and complete occlusion, was present in various studies, but that each considered one, two or the three types of occlusion for its outcome. The present study considered occlusion without reflux and complete occlusion as the outcome. In addition, in the studies found there is no homogeneity of heparin concentration, volume of solution, type of CVC and frequency of the permeability test.

## Conclusion

The catheters allocated for blocking with heparin solution showed better performance compared to those allocated for blocking with ISS. Therefore, the hypothesis was accepted that blocking with a 50 IU/mL heparin solution is more effective than ISS in preventing occlusion of the Hickman^®^ catheter in patients undergoing HSCT.

Further clinical trials are recommended to investigate the effectiveness of heparin in maintaining the permeability of the Hickman^®^ catheter, using the same methodological design in other populations.
